# The influence of *Osmunda regalis* root extract on head and neck cancer cell proliferation, invasion and gene expression

**DOI:** 10.1186/s12906-017-2009-4

**Published:** 2017-12-04

**Authors:** Marianne Schmidt, Josef Skaf, Georgiana Gavril, Christine Polednik, Jeanette Roller, Michael Kessler, Ulrike Holzgrabe

**Affiliations:** 1 Department of Otorhinolaryngology, University of Wuerzburg, Josef-Schneider-Straße 11, D-97080 Wuerzburg, Germany; 2 Institute of Pharmacy and Food Chemistry, University of Wuerzburg, Am Hubland, 97074 Würzburg, Germany; 3 Biological Research Centre, Laboratory of Vegetal Biology, 6 Alexandru cel Bun street, Piatra Neamţ, Romania

**Keywords:** HNSCC, Head and neck carcinoma, Plant extract, Proliferation, Invasion, Metastasis, Gene expression, *Osmunda regalis*

## Abstract

**Background:**

According to only a handful of historical sources*, Osmunda regalis,* the royal fern, has been used already in the middle age as an anti-cancer remedy. To examine this ancient cancer cure, an ethanolic extract of the roots was prepared and analysed in vitro on its effectiveness against head and neck cancer cell lines.

**Methods:**

Proliferation inhibition was measured with the MTT assay. Invasion inhibition was tested in a spheroid-based 3-D migration assay on different extracellular matrix surfaces.

Corresponding changes in gene expression were analysed by qRT-PCR array. Induction of apoptosis was measured by fluorescence activated cell sorting (FACS) with the Annexin V binding method. The plant extract was analysed by preliminary phytochemical tests, liquid chromatography/mass spectroscopy (LC-MS) and thin layer chromatography (TLC). Anti-angiogenetic activity was determined by the tube formation assay.

**Results:**

*O. regalis extract* revealed a growth inhibiting effect on the head and neck carcinoma cell lines HLaC78 and FaDu. The toxic effect seems to be partially modulated by p-glycoprotein, as the MDR-1 expressing HLaC79-Tax cells were less sensitive. *O. regalis extract* inhibited the invasion of cell lines on diverse extracellular matrix substrates significantly. Especially the dispersion of the highly motile cell line HlaC78 on laminin was almost completely abrogated.

Motility inhibition on laminin was accompanied by differential gene regulation of a variety of genes involved in cell adhesion and metastasis. Furthermore, *O. regalis extract* triggered apoptosis in HNSCC cell lines and inhibited tube formation of endothelial cells. Preliminary phytochemical analysis proved the presence of tannins, glycosides, steroids and saponins. Liquid chromatography/mass spectroscopy (LC-MS) revealed a major peak of an unknown substance with a molecular mass of 864.15 Da, comprising about 50% of the total extract. Thin layer chromatography identified ferulic acid to be present in the extract.

**Conclusion:**

The presented results justify the use of royal fern extracts as an anti-cancer remedy in history and imply a further analysis of ingredients.

## Background

Head and neck squamous cell carcinoma (HNSCC) is one of the leading causes of cancer death worldwide. According to an analyis in 2009 of over 3000 cases of primary head and neck tumours in Germany 2009, the outcome has not improved significantly from 1995 to 2006, despite new treatment strategies. Especially the 5-year overall survival rate for carcinomas of hypopharyngeal origin is very low with 27.2% [1]. The cure of head and neck cancer is predominantly influenced by the stage of metastasis and the mode of invading the surrounding environment (for review see [2]). HNSCC predominantly metastasize into locoregional lymph nodes rather than to other organs.

Combined chemotherapy and radiation is meanwhile commonly used for advanced head and neck cancer in order to preserve laryngeal and/or pharyngeal structures. Paclitaxel is one of the agents used with high response rates, however it failed to reach a local-regional tumour control in 12% of patients according to a previously published study [3].

*Osmunda regalis*, the royal fern, is a largely forgotten medical plant. One very early source for the use of *Osmunda regalis* roots in the treatment of ulcers originates from the middle-age surgeon Hieronymus Brunschwig. “Das kleine Destillierbuch” was published in 1500 in Straßburg [4]. Furthermore *Osmunda regalis* was mentioned in Jonathan Hartwells compendium “Plants used against Cancer” [5], where he refers to a publication in 1849 of S.W. Williams on indigenous medicinal plants of Massachusetts [6].

In 2011 Toji Thomas [7] proved an anti-bacterial effect of diverse extracts of *Osmunda* leaves. *Osmunda regalis* as an anti-cancer phyto-medicine has fallen into oblivion. No further investigations on this plant have been published to our knowledge. In this study we analyzed the influence of an *Osmunda regalis* ethanolic root extract on growth, behaviour and gene expression in head and neck cancer cell lines.

## Methods

### Cell lines and cell culture

The cell line FaDu originating from a hypopharyngeal carcinoma was grown with RPMI 1640 medium (Seromed, Munich, Germany), supplemented with 10% fetal calf serum (FCS). HLaC78 cell line originated from a larynx carcinoma [8] and was kept as FaDu in RPMI 1640 Medium. HLaC79 (larynx carcinoma, see above) cells were treated with 10 nM Paclitaxel. A taxol-resistant clone was isolated by selective trypsination of single clones. The permanent HLaC79 clonal cell line HLaC79-Tax was cultured in RPMI 1640 medium, supplemented with 10% FCS and 10 nM Paclitaxel.

### *Osmunda regalis* ethanolic extract

**Fig. 1:**
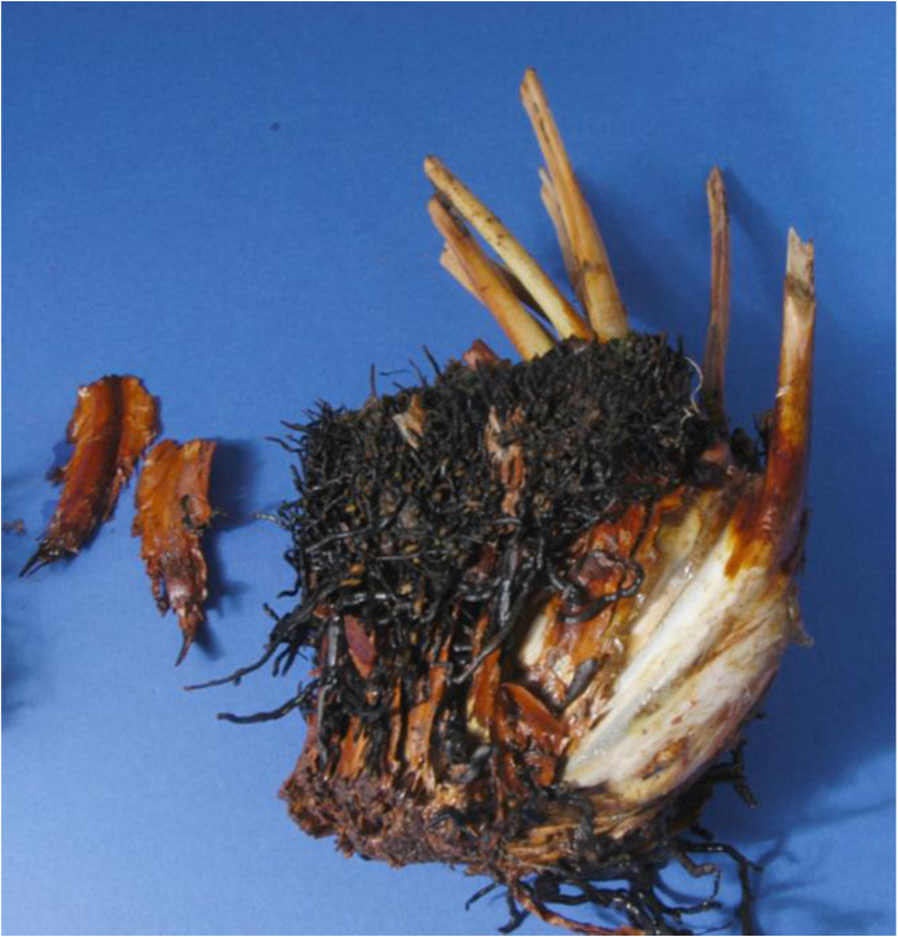
Bulb of Osmunda regalis, composed of wooden sheets and black roots

*Osmunda* plants originated from the Botanical Garden of the University of Kaiserslautern (Germany). They were identified by Mr. Bernd Simon, who is a known expert for plant taxonomy. A voucher specimen was deposited at the Herbarium of the University of Wuerzburg; (Index Herbariorum Code: WB) under the number 2017_HNO001. The black roots (Fig. 1) were cleaned, dried and minced. The ethanolic extract was prepared as follows: 18.5 g of minced roots were homogenized in 30 ml 70% ethanol with a power homogenizer and subsequently agitated overnight at 37 °C. After 14 days incubation with daily agitation, the supernatant was cleared by centrifugation and sterile filtration. The yield after centrifugation and sterile filtration was 20 ml. A 1 ml aliquot of the extract was dried by centrifugal evaporation. According to the weight of the dried substance the concentration of the extract was adjusted to 6 mg/ml with 70% ethanol. Aliquots of the stock solution were stored at −80 °C. For experiments the stock solution was diluted 1:10 with culture medium without supplements (0.6 mg/ml). This working solution was finally diluted to 6, 15, 30, 60 and 90 μg/ml for MTT assays. One batch was used for all experiments.

### Preliminary phytochemical tests

Phytochemical tests were performed as described [9, 10]. Tests used are summarized in Table 1.

**Table 1:** Tests used for preliminary phytochemical screening

Phytochemical class	Tests used for analysis
Tannins	Braymer’s Test [10]
Flavonoids	Shinoda Test [10]
Saponins	Foam Test [10]
Alkaloids	Hager’s Test [9]
Glycosides	Salkowski’s Test [10]
Steroids	Liebermann-Burchard test [10]
Terpenoids	Terpenoid Test [10]

### LC/MS analysis and MS data

LC-MS analysis was performed using a Shimadzu LC-MS-2020 mass spectrometer (Shimadzu Deutschland GmbH; Duisburg, Germany) containing a DGU-20A3R degassing unit, a LC20AB liquid chromatograph and SPD-20A UV/Vis detector. As stationary phase a Synergi 4 U fusion-RP column (150 × 4.6 mm; Phenomonex, Aschaffenburg, Germany) and as mobile phase a gradient of MeOH/water was used.

Parameters for the method: Solvent A: water with 0.1% formic acid, solvent B: MeOH with 0.1% formic acid. Solvent A from 0% to 100% in 8 min, then 100% for 5 min, from 100% to 5% in 1 min, then 5% for 4 min, The method was performed with a flow rate of 1.0 mL/min, UV detection was measured at 245 nm.

### TLC analysis/sample identification

As samples for TLC, *O. regalis extract* and standards were used. They were applied to Silica gel 60 F264 plates (Merckmillipore.com), according to standard procedures [11]. As solvents ethyl acetate - formic acid - acetic acid - water (100:11:11:27) for flavones or toluene - ethyl acetate - formic acid (50:40:10) for polyphenolcarboxylic acids were used. UV detection was performed at 365 nm.

The following standards were used: ferulic acid, apigenin (Extrasynthese, Genay, France), chlorogenic acid (Carl Roth, Rothenfels, Germany), rosmarinic acid and rutoside (Sigma Aldrich, St. Louis, Missouri, USA).

### Cell viability and proliferation assay

Cells were seeded at 5000 cells/well in 96 well plates. *O. regalis* ethanolic extract (6 mg/ml) was diluted 1:10 with cell culture medium without supplements and subsequently further diluted. Cells were treated with increasing concentrations (0, 6, 15, 30, 60, 90 μg/ml) of *Osmunda regalis* extract for 48 h. After 48 h cell culture medium was removed and replaced by medium supplemented with MTT (1 mg/ml) [12]. Following 4 h incubation, isopropanol replaced medium for 45 h at 37 °C. Colour conversion of the MTT reaction was measured at a wavelength of 570 nm. Relative toxicity was calculated as % surviving cells by setting control cells treated with vehicle as 100% surviving cells. Viability was calculated to the following formula: absorption Control cells (untreated)/100 * absorption treated cells.

### Apoptosis

Apoptosis was measured with an annexin V-based test [13]. Annexin V binds to phosphatidylserin, which is usually exposed on the inner surface of the plasma membrane. As an early event of apoptosis it is translocated to the outer surface. To differentiate between vital, apoptotic and necrotic cells, DNA-binding propidium iodide (PI), which is unable to pass the cell membrane, was used. Annexin V and PI positive cells indicate cells in the end stage of apoptosis or at necrotic/undefined cell death. FACS analysis was performed with a FACS automated system (BD FACSCanto, BD, Heidelberg, Germany), using the Annexin V-APC kit of BD Pharmingen (BD Biosciences, Heidelberg, Germany) according to the kit manual. In brief, HLaC78 and FaDu cells were treated with the EC_50_ concentrations for 24 h, harvested and washed twice with cold PBS. Cells were then resuspended in 1 x binding buffer (0.1 M Hepes, pH 7.4, 1.4 M NaCl, 25 mM CaCl_2_) at a concentration of 10^6^ cells/ml. To 100 μl of this cell suspension 5 μl Annexin V-APC and 5 μl 7-AAD (included in the kit) were added, the cells were vortexed and incubated for 15 min in the dark. 400 μl of 1 x binding buffer was added. Within one hr. FACS analysis was performed at an excitation wavelength of 650 nm. Cells were analyzed by the corresponding Software (FACSDiva, BD, Heidelberg, Germany).

### In vitro motility assays

Tumour spheroids were generated by seeding 5000 cells/well of HLaC78 and FaDu cells on ultra-low-attachment (ULA) 96-well round-bottomed plates (Corning, Amsterdam, Netherlands) [14]. For the migration assay spheroids of HLaC78 and FaDu were placed on different extracellular matrix substrates [15]. The surface of flat-bottomed 96-well plates were coated with 0.1% Gelatin, 5 μg/ml Fibronectin, 50 μg/ml Laminin, 50 μg/ml Collagen I (all from Sigma Aldrich, Taufkirchen, Germany) or 125 μg/ml Matrigel® (Becton Dickinson, Heidelberg, Germany) for 2 h at room temperature. Wells were washed twice with PBS and subsequently blocked with 1% bovine serum albumin in PBS for 1 h. On ULA plates for 2 days cultivated spheroids of both cell lines were transferred to the coated wells with a multichannel pipette. Spheroids were incubated with or without *O. regalis extract* (EC_50_). Migration was recorded by photographing spheroids after 1 and 24 h with a Leica DMI 4000 inverted fluorescence microscope (Leica Microsystems, Wetzlar, Germany). Quantification of migrated cells was carried out with the ImageJ software (National Institutes of Health, NIH, USA).

### RNA extraction and RNA quality control

RNA was isolated with the RNeasy kit (Qiagen, Hilden, Germany) according to the manufacturer’s instructions. RNA quality was assessed with the RNA 6000 nano kit using the Bioanalyzer 2100 instrument (Agilent, Böblingen, Germany). RNA integrity numbers (RINs) of the samples ranged between 9.4 and 9.9.

### Taqman PCR-Array and Taqman qRT-PCR

Expression changes were analysed by quantitative Taqman-RT-PCR [16]. For analysis of expression changes caused by *O. regalis* extract in invading cells, HLaC78 spheroids were grown and transferred to laminin coated 96 well plates as described above. After 18 h invasion with or without plant extract, spheroids and invaded monolayer cells were harvested by trypsination and pooled. RNA was isolated (see above). To evaluate gene expression changes, Taqman array plate for cell adhesion molecules (Applied Biosystems, Darmstadt, Germany) were supplied with cDNA, reverse transcribed with the high capacity RNA to cDNA Master Mix (Applied Biosystems, Darmstadt, Germany) according to the manufacturers protocol. Taqman qRT-PCR was carried out according to the manufacturer’s instructions for MDR-1.

### Tube formation assay

Using tube formation assays, the ability of endothelial cells to form three-dimensional capillary-like structures was analyzed.

Ibidi angiogenesis-slides (15-well, Ibidi GmbH, Munich, Germany) were coated with growth factor reduced basement membrane extract (BME; Trevigen, MD, USA.). After polymerization of BME, the gels were overlaid with growth medium containing 10^4^ HUVEC and 8 μg/ml *Osmunda regalis* extract. Cells were incubated for 6 h and images were taken. Evaluation of pictures was performed by Wimasis GmbH (Munich, Germany). For quantification, four parameters were analyzed: tube length, number of branching points, covered area and number of loops.

### Statistical analysis

All statistical analyses and graphs were performed with Graph Pad Prism 6 (Graphpad Software, La Jolla, USA).

## Results

### Phytochemical analysis of the extract

Due to historical sources it wasn’t clear, which parts of the plant had been used formerly. Williams et al. [6] used the “bulb” for production of an alcoholic extract. The bulb of *Osmunda* fern plants is composed of a hard wooden splintery part, surrounded by black roots (Fig. 1). In a first attempt (data not shown) we tried to extract active ingredients from the wooden parts by shredding and extracting them in 70% ethanol for prolonged periods. The preliminary MTT results, however, were disappointing. Unlike the wooden bulb, the black roots proved to be much more promising.

Preliminary phytochemical tests of the extract revealed a quick overview over the substance classes, present in *Osmunda* root extract.

According to Braymer’s Test the ethanolic extract was clearly positive for tannins, indicated by a dark green-blue staining. The foam test pointed to the presence of saponins. Furthermore a red staining in the chloroform layer of the Liebermann-Burchard test confirmed the presence of steroids. In contrast neither alkaloids, flavonoids or terpenoids could be detected with Hager’s test, Shinoda test or terpenoid test, respectively. Results are summarized in Table 2.

**Table 2:** Preliminary phytochemical Tests of ethanolic Osmunda extract

Tannins	**+**
Glycosides	**+**
Alkaloids	**–**
Steroids	**+**
Flavonoids	**–**
Saponins	**+**
Terpenoids	**–**

### LC-MS analysis

**Fig. 2:**
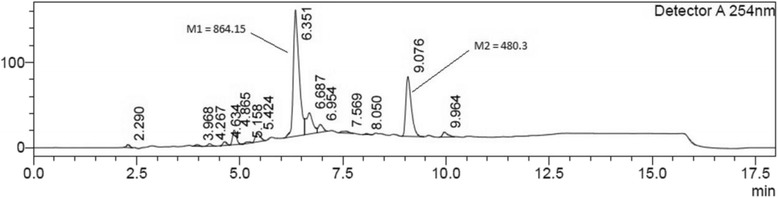
UV-chromatogram of Osmunda regalis ethanolic extract

LC-MS analysis of the *Osmunda regalis* ethanolic root extract displayed two major peaks at 6.3 min and 9.1 min in the UV chromatogram at 254 nm (Fig. 2) with the percentage area of 50.8% and 22.9% and the corresponding masses of 864.15 (M1) and 480.3 (M2) respectively (Fig. 2). All LC-MS peaks are displayed in Table 3.

**Table 3:** LC-MS peaks of *Osmunda regalis* ethanolic extract

Peak#	Ret.Time	Area	Height	Area %
1	2.290	18,595	3366	0.662
2	3.968	12,510	1821	0.446
3	4.267	19,559	2826	0.697
4	4.634	28,091	4357	1.001
5	4.865	111,327	17,033	3.965
6	5.158	15,513	1831	0.553
7	5.424	85,522	9706	3.046
8	6.351	1,427,094	147,461	50.831
9	6.687	291,579	24,799	10.386
10	6.954	73,496	9117	2.618
11	7.569	24,235	2083	0.863
12	8.050	6293	1035	0.224
13	9.076	644,082	69,768	22.941
14	9.964	49,623	5676	1.768
Total		2,807,521	300,882	100.000

### Sample analysis by thin layer chromatography (TLC)

**Fig. 3:**
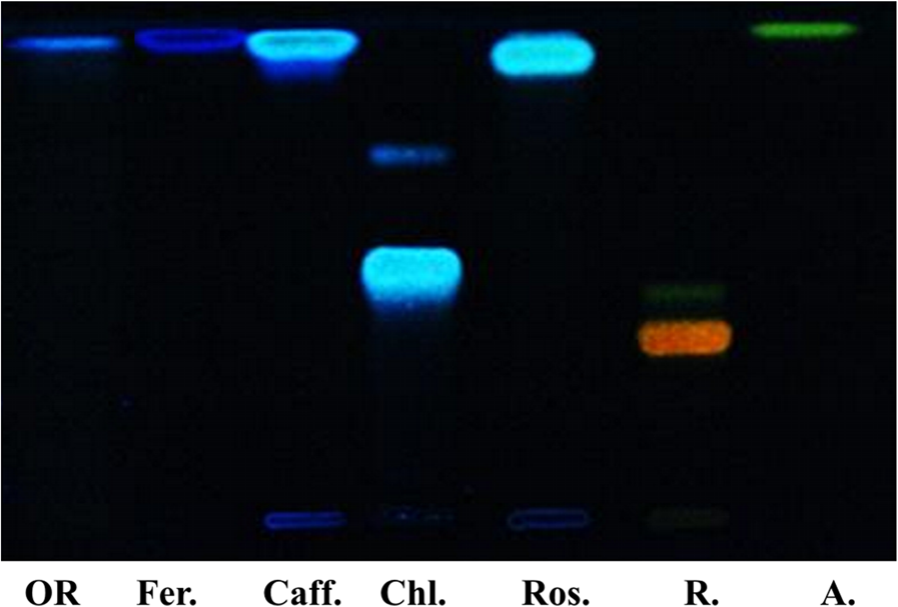
TLC chromatogram for polyphenolic acids and flavonoids of Osmunda regalis ethanolic extract: Osmunda regalis ethanolic extract (1), Standards: ferulic acid (Fer.), caffeic acid (Caff.), chlorogenic acid (Cl.), rosmarinic acid (Ros.), rutoside (R.), apigenin (A.)

Qualitative phytochemical analysis of polyphenolic acids present in the *Osmunda regalis* extract was performed by thin layer chromatography. The chromatographic image with polyphenolcarboxylic acid standards revealed a single blue band, which corresponded to ferulic acid (Fig. 3).

### Cytotoxicity

**Fig. 4:**
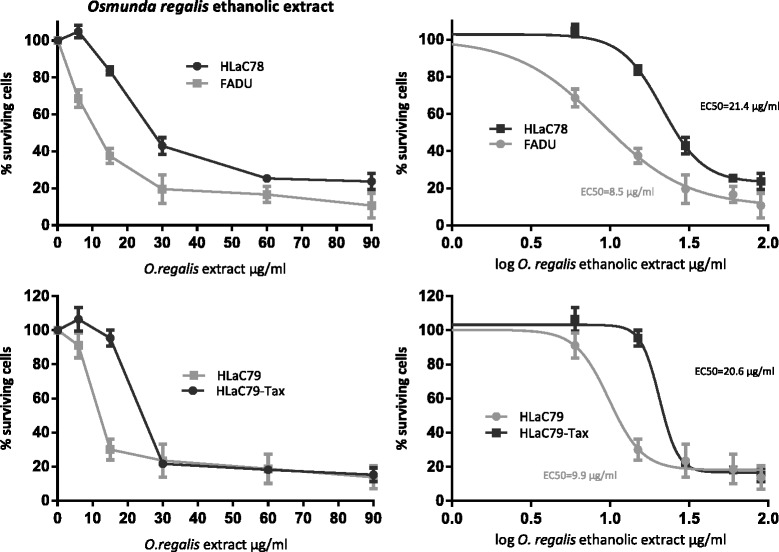
Cytotoxicity of Osmunda regalis ethanolic extract in increasing concentrations on HNSCC cell lines HLaC78 and FADU, and HLaC79/HLaC79-Taxdetermined by MTT assay. Calculated EC50 Doses were 0.85µg/ml or 2.14 µg/ml for FADU or HLaC78 and 0.99 or 2.14 µgl/ml for HLaC79 and HLaC79-Tax, respectively, after 48 h incubation

**Fig. 5:**
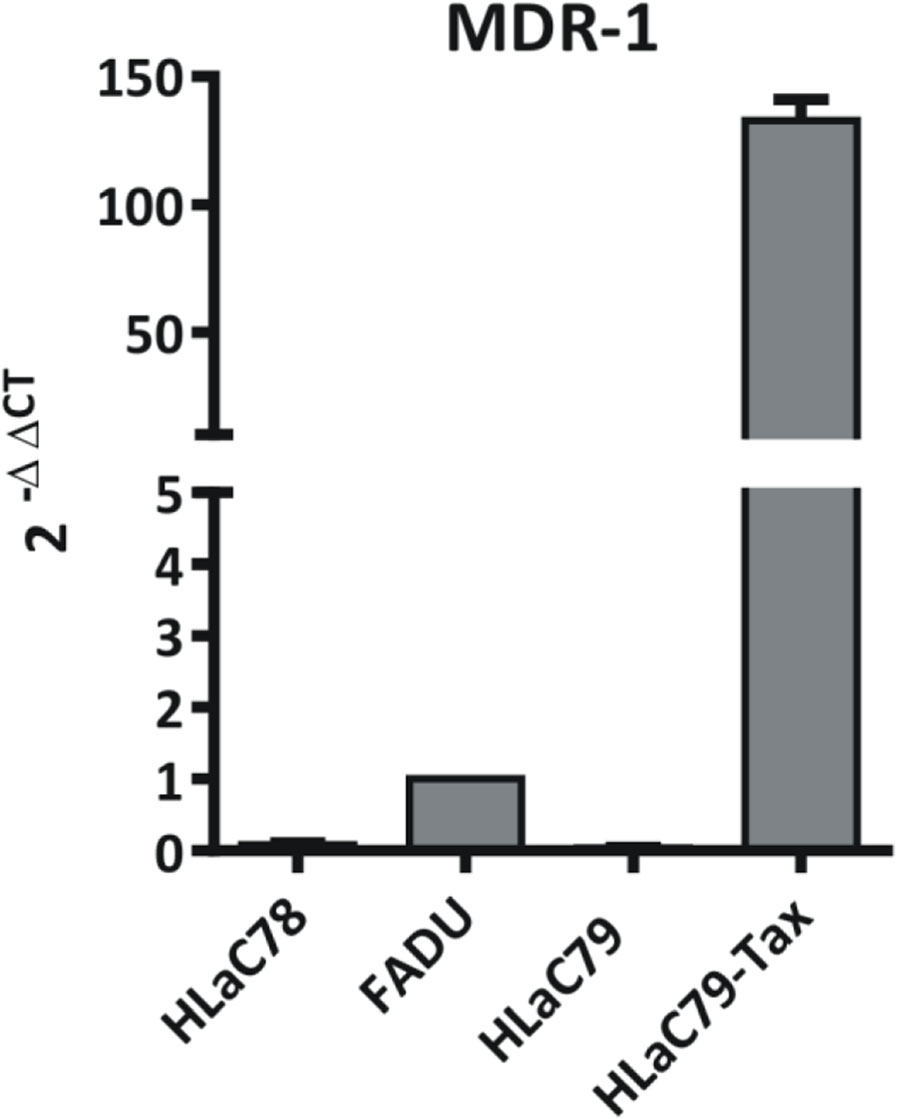
Expression of MDR-1 mRNA in HLaC78, HLaC79, HLaC79-Tax and FADU

The cell lines FaDu and HLaC78 were treated with increasing concentrations of *O. regalis extract*. Cell viability and cytotoxicity were quantified with the MTT assay (Fig. 3). Mean percentage inhibition was calculated from at least three independent experiments. *Osmunda regalis* extract significantly suppressed the growth of HLaC78, FaDu, HLaC79 and HLaC79-Tax cell lines with increasing concentrations (1way ANOVA, *p* < 0.0001; Fig. 4). Mean effective concentration (EC_50_) was higher in HLaC78 cells (21.4 μg/ml) than in FaDu cells (8.5 μg/ml), although HLaC78 resistance is not based on expression of p-glycoprotein (Fig. 5). HLaC79-Tax is a descendant cell line of HLaC79 with acquired resistance against paclitaxel and corresponding over-expression of p-glycoprotein [17]. Here, the EC_50_ dose of *O. regalis extract* was higher in HLaC79-Tax (20.6 μg/ml) compared to the parental cell line HLaC79 (9.9 μg/ml).

Expression of MDR-1 (coding for p-glycoprotein) is displayed in Fig. 5.

### Apoptosis

**Fig. 6:**
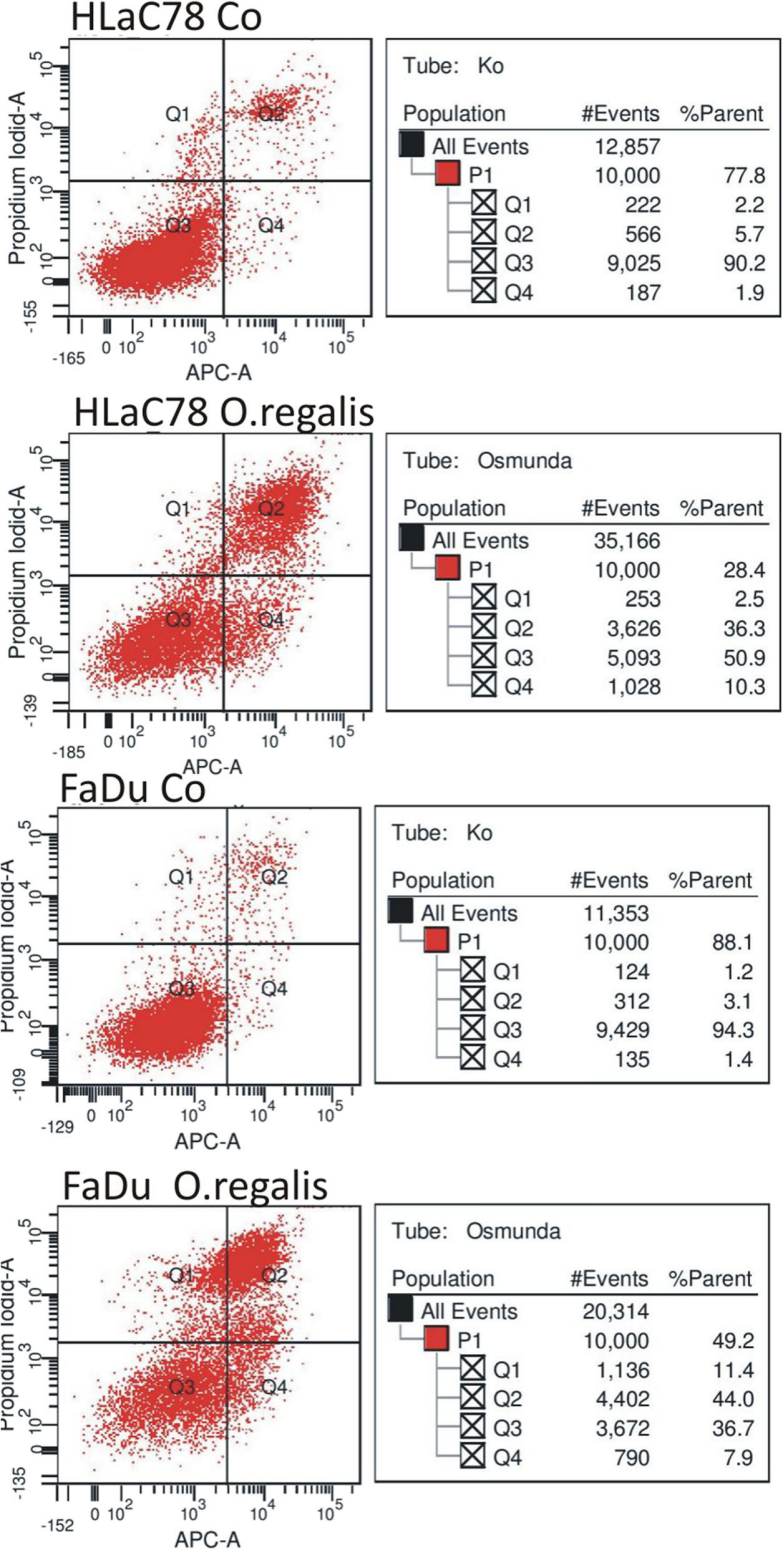
FACS analysis with the Annxin V-Test of HLaC78 and FADU, treated with EC50 of Osmunda regalis ethanolic extract. Q1: PI positive, Annexin V negative cells; necrotic or undefined cell death; Q2: Annexin V positive, PI positive cells, cells in late apoptosis; Q3: Annexin V negative, PI negative cells; vital cells Q4: Annexin V positive, PI negative; pre-apoptotic cells. Ko= untreated control cells.

*Osmunda regalis* ethanolic extract clearly induced apoptosis in both cell lines at their EC_50_ doses, as measured by FACS analysis with the AnnexinV-Test (Fig. 6). In FaDu the extract revealed 7.9%, in HLaC78 10.3% early apoptotic stages after 24 h incubation with *O. regalis extract*. Results of the apoptosis assay are summarized in Fig. 6.

### Cell motility on extracellular matrix (ECM) proteins

Investigation of invasion and motility was carried out using spheroid-based experiments. In contrast to the commonly used Boyden Chamber assay this kind of invasion measurement proved to be reliable and reproducible.

Spheroids of both cell lines were grown in ultra-low-attachment-plate (ULA-plate) wells and subsequently transferred manually to wells, coated with different ECM substrates: Gelatin, Fibronectin, Laminin, Collagen I and Matrigel® and treated with or without *Osmunda regalis* aqueous extract. Photographs of the cells were taken after attachment to ECM (1 h, *t* = 0) and after 18 h (*t* = 18).

For quantification of cells migration the areas of spheroids at *t* = 0 and *t* = 18 were photographed and outgrown areas were measured with ImageJ software (area calculation). For each condition (with or without *Osmunda*, HLaC78 or FaDu) 8 spheroids were measured.

For evaluation of cell motility the spheroid area at *t* = 0 was set at 100% and the percentage of invaded area was calculated.

**Fig. 7:**
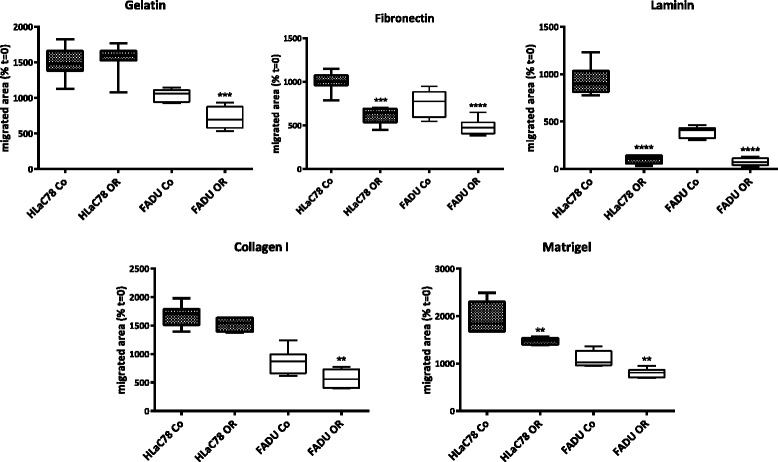
Inhibition of substrate invasion by Osmunda ethanolic extract (0.85 µg/ml FADU or 2.14µg/ml HLaC78; 18 h). HLaC78 and FADU cell lines were tested with (OR) or without O. regalis (Co) extract on Gelatin-, Fibronectin-, Laminin-, CollagenI- and Matrigel®-coated surfaces. Statistically significant values are marked with asterisks (unpaired t-test; ** *p*<0.01, *** *p*<0.001, **** *p*<0.0001)

*O. regalis extract* significantly inhibited invasion of highly invasive HLaC78 cells on fibroectin, laminin and matrigel. Invasion of FaDu cells was significantly inhibited on all substrates (Fig. 7).

**Fig. 8:**
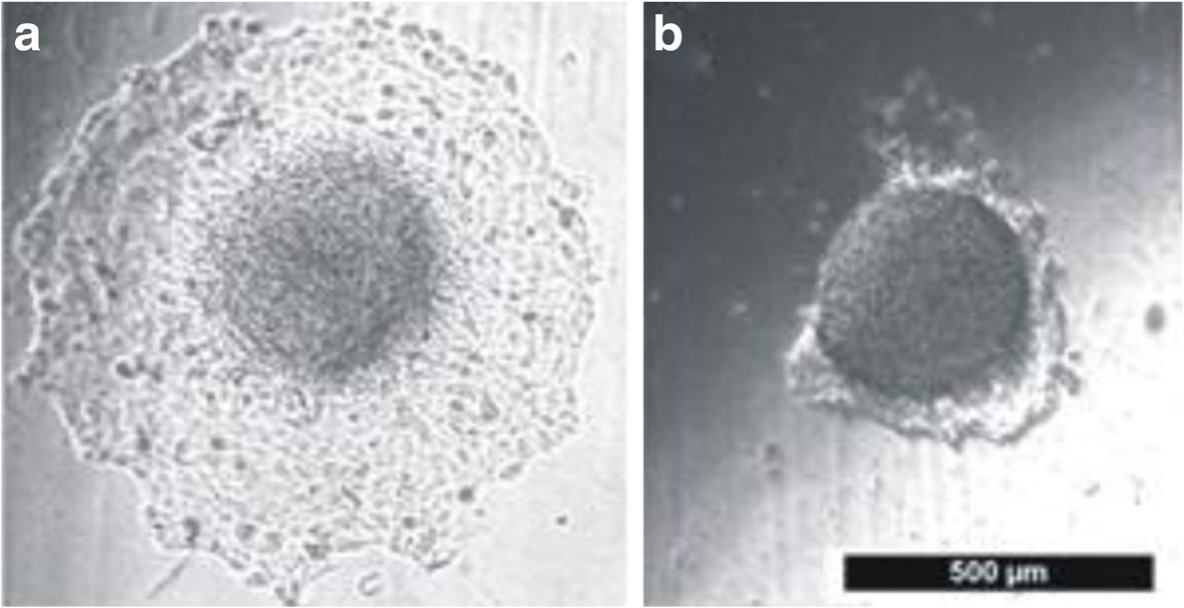
Invasion pattern of HLaC78 cells on laminin coated surface after 18 h incubation with (**b**) or without (**a**) O, regalis extract

A representative example for HLaC78 cells migrating on laminin with or without *O. regalis* extract is shown in Fig. 8.

### Gene expression

For analysis of expression changes caused by *O. regalis extract* in strongly invading HLaC78 cells, a spheroid-based invasion assay on laminin, which showed the strongest inhibition in both cell lines was performed. After 18 h invasion time, spheroids with or without *O. regalis extract* (2.14 μg/ml) were harvested and RNA was isolated. To analyse gene expression changes, Taqman qRT-PCR array plates for metastasis and cell adhesion molecules (Applied Biosystems, Darmstadt, Germany) were used.

**Fig. 9:**
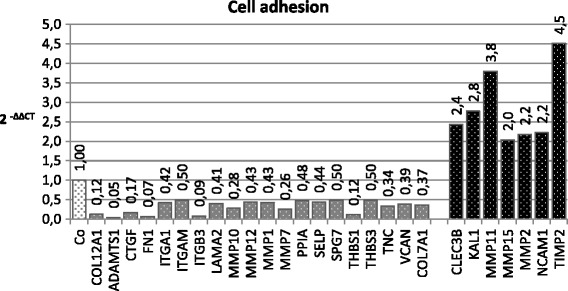
Expression changes in HLaC78 cells invading on laminin, caused by O. regalis extract after 18 h invasion time. Expression increases ≥ 2 and decreases ≤ 0.5 are displayed

Gene expression analysis of both PCR arrays in invading HLaC78 cells revealed a significant up−/downregulation of the following genes upon incubation with *O. regalis extract* (Fig. 9).

According to the taqman cell adhesion array 7 genes were significantly up-regulated (Fig. 9): CLEC3B (coding for tetranectin), KAL1 (anosmin-1), MMP11, 15, 2 (matrix metalloproteinases), NCAM1 (neural cell adhesion molecule) as well as TIMP2 (coding for tissue inhibitor of MMP2). 20 genes were significantly down-regulated (Fig. 9), among them several integrins (ITGA1, ITGAM, ITGB3), proteases (ADAMTS1, MMP1, 7, 10, 12) and further metastasis-relevant genes (CTGF – connective tissue growth factor, PPIA – cyclophilin A, SELP – P-selectin, TNC – tensasin C, THBS1 – thrombospondin 1, VCAN – versican).

### Angiogenesis

**Fig. 10:**
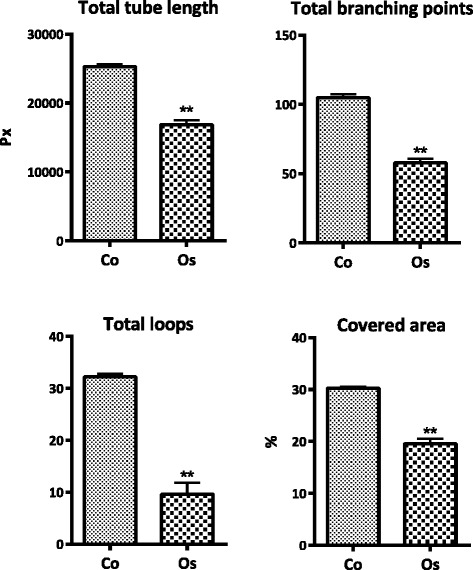
Analysis of angiogenesis with the tube formation assay. HUVEC tube formation was measured in untreated endothelial cells (Co) and endothelial cells treated with O. regalis extract (0.85µg/ml). For evaluation total tube length (measured in pixels px), number of branching points, number of total loops and percentage covered area (%) were measured

Effects of *O. regalis extract* on angiogenesis were tested with the tube formation assay. Total tube length, total branching points, total loops and % covered area were quantified and revealed a statistical significant decrease of all tube formation parameters upon treatment with *O. regalis extract* (8.5 μg/ml; Fig. 10).

## Discussion

*Osmunda regalis,* the royal fern has fallen into oblivion as a traditional medical plant. Only a few sources mentioned this plant for the cure of ulcers in history [4–6].

In the present study the effect of an ethanolic *Osmunda regalis* root extract was tested on different head and neck cancer cell lines.

According to a combined HPLC/mass spectroscopy *O. regalis* extract showed two major peaks at the masses of 864.15 and 480.3 Da., as well as minor peaks at 464.3, 480.3, 288.05, 307.1, 325.05, 616.1 and 505.5 Da.

The masses 464 and 480 probably correspond to the antifeedant insect hormones ecdysone and ecdysterone/ponasterone A, which have been described to be present in *Osmunda* japonica (Medicinal Plant Images Database of the Baptist University of Hongkong. (http://library.hkbu.edu.hk/electronic/libdbs/mpd/). Most abundant is an unknown substance with a mass of 864.15, comprising about 50% of total ingredients.

TLC analysis identified only one band in the extract, corresponding to ferulic acid.

*O. regalis extract* revealed a distinct growth inhibition in all cell lines. The significantly higher EC_50_ in the HLaC79-Tax cell line, overexpressing MDR-1, suggests the involvement of p-glycoprotein in the elimination of active ingredients. The increased resistance of HLaC78, however, must be based on (a) different drug resistance mechanism(s) [18] against the ingredients of the *O. regalis* extract. An increased resistance of HLaC78 has never been observed before with plant extracts [19] or chemotherapeutics (data not shown).

In addition to growth suppression *O. regalis extract* strongly inhibited cell migration on diverse ECM substrates, even in the highly invasive HLaC78 cell line. Invasion was most distinctly inhibited on laminin-coated surfaces. Compared to gelatin, fibronectin, collagen or matrigel, migration on laminin was nearly abandoned in both cell lines by *O. regalis extract*. Ferulic acid might be at least partially responsible for the anti-invasive properties of the extract, as it has been reported to act anti-invasive in breast cancer in vitro models [20].

Since HNSCC invasion comprises a finely tuned interaction network of factors involved in ECM production (such as laminins, collagens), degradation (proteases) and cell adhesion (integrins), the three-dimensional spheroid invasion model was chosen for the analysis of gene expression, involved, when cells are leaving the tight tumour-like tissue. Thus gene expression during spheroid invasion on laminin-coated surfaces with or without *O. regalis extract* has been compared with respect to cell adhesion related genes. Several genes involved in HNSCC invasion such as matrix metalloproteinases, laminin and integrins were down-regulated by *O. regalis extract*. According to the review of Iizuka et al. [21], evaluating expression profiling studies in HNSCC, matrix metalloproteinases MMP-1, −3, −7, −10, −12 and −13 correlate with HNSCC tumorigenicity and progression. Here MMP-1, 7, 10 and 12 were down-regulated by *O. regalis* extract. ADAMTS1 (coding for a Disintegrin and Metalloprotease with Thrombospondin motifs) was down-regulated by *O. regalis extract*. High expression rates have been associated with tumor progression, metastasis and impaired survival in vivo in different tumor systems (for review see [22]). Moreover ADAMTS1 and MMP1 play a role in bone metastasis of breast cancer cells [23]. Kuang et al. [24] identified FN1 (fibronectin), which was strongly suppressed by *O.regalis* extract, as one of the major up-regulated genes in head and neck cancer specimen.

PPIA, a further gene down-regulated by *O. regalis extract* and coding for Cyclophilin A, has been related to tumor progression and/or metastasis of a variety of cancer types (reviewed in [25]). Expression of THBS1, coding for Thrombospondin1 was diminished by *Osmunda*. This protein seems to play a role in epithelial-mesenchymal transition (EMT), it has been shown to be up-regulated in melanoma cells of the most mesenchymal phenotype [26]. The role of the thrombospondins in cancer and metastasis however, is not clearly defined yet. There are several examples, where TSBH1 is responsible for enhanced growth or invasion, but also cancer systems in which TSBH1 had an inhibitory effect (reviewed in [27]). The ECM glycoprotein tenascin C (gene locus TNC) has been shown to correlate strongly with metastasis. It promotes migration and invasion and high expression of TNC has been shown to predict poor clinical outcome in head and neck cancer [28]. TNC was strongly down-regulated by *O. regalis extract*. Similarly the proteoglycan versican, coded by the gene VCN seems to be involved in tumor progression and metastasis, as has been shown for renal cell carcinoma [29]. CLEC3B is one among the few genes, up-regulated by *Osmunda*, coding for tetranectin. This lectin has been shown to be under-expressed in both serum and saliva of metastatic oral squamous cell carcinoma (OSCC) compared to primary OSCC [30].

In addition to growth and invasion inhibition angiogenesis was significantly inhibited by *Osmunda regalis* extract. Due to the nature of the tube formation assay this result is based on the effect of the extract on endothelial cells and doesn’t provide information about angiogenesis in its overall context, implying the vascular endothelial growth factor (VEGF) pathway. Interestingly Yang et al. reported, that ferulic acid inhibits angiogenesis by targeting the FGFR1-mediated (fibroblast growth factor-1) PI3K-Akt signaling pathway in endothelial cells [31], which might explain the anti-angiogenic properties of the *O.regalis* extract.

## Conclusions

Summarizing the actual results *Osmunda regalis* ethanolic extract revealed a growth inhibiting effect on the cell lines HlaC78 and FaDu. Toxic effects seem to be partially modulated by p-glycoprotein, as the strongly MDR-1 expressing HLaC79-Tax cells are less sensitive to *O. regalis extract*. *O. regalis extract* inhibited dispersion of HlaC78 and FaDu cells on ECM -coated surfaces significantly, most pronounced in the highly motile cell line HlaC78 on laminin. The observed motility inhibition was accompanied by gene expression modulation of a variety of genes concerning cell adhesion and metastasis. *O. regalis* extract triggered apoptosis in HNSCC cell lines and inhibited tube formation of endothelial cells. All these features of *Osmunda* reported here, justify the use of *Osmunda regalis* as an anti-cancer remedy in the past. Further examination and identification of substances is indicated.

## Abbreviations

7-AAD: 7-aminoactinomycin D
Annexin V-APC: Annexin V Allophycocyanin conjugate
ECM: extracellular matrix
FACS: fluorescence activated cell sorting
FCS: fetal calf serum
HNSCC: head and neck squamous cell carcinoma
LC-MS: liquid chromatography mass spectroscopy
MDR-1: multi drug resistance gene 1 (p-glycoprotein)
MTT: 3-(4,5-Dimethylthiazol-2-yl)-2,5-diphenyltetrazoliumbromid)
OSCC: oral squamous cell carcinoma
PBS: phosphate buffered saline
PI: propidium iodide
qRT-PCR: real time quantitative PCR
TLC: thin layer chromatography
ULA: ultra-low attachment

## References

[1] Guntinas-Lichius O, Wendt T, Buentzel J, Esser D, Lochner P, Mueller A, Schultze-Mosgau S, Altendorf-Hofmann A (2010). Head and neck cancer in Germany: a site-specific analysis of survival of the Thuringian cancer registration database. J Cancer Res Clin Oncol.

[2] Harrison LB, Sessions RB, Kies MS. Head and neck cancer: a multidisciplinary approach. 4th ed: Lippincott Williams and Wilkins; 2013.

[3] Pfreundner L, Hoppe F, Willner J, Preisler V, Bratengeier K, Hagen R, Helms J, Flentje M (2003). Induction chemotherapy with paclitaxel and cisplatin and CT-based 3D radiotherapy in patients with advanced laryngeal and hypopharyngeal carcinomas--a possibility for organ preservation. Radiother Oncol.

[4] Will H: Vergleich der Indikationen des ‚Kleinen Destillierbuches’ des Chirurgen Hieronymus Brunschwig (Straßburg 1500) mit den nach derzeitigem wissenschaftlichem Erkenntnisstand belegten Indikationen. Wuerzburg: Julius-Maximilians-Universität Würzburg; 2009.

[5] Hartwell JL. Plants used against cancer. Massachusetts USA: Quarterman Publications, Inc.; 1982.

[6] Williams S (1849). Report on the indigenous medical botany of Massachusetts. Trans Amer Med Assoc.

[7] Thomas T (2011). Preliminary antibacterial and phytochemical assessment of Osmunda Regalis L. Int J Pharmaceut Biol Arch.

[8] Zenner HPLW, Herrmann IF (1979). Establishment of carcinoma cell lines from larynx and submandibular gland. Arch Otorhinolaryngol.

[9] Yadav MCS, Gupta SK, Watal G (2014). Preliminary phytochemical screening of six medicinal plants used in traditional medicine. Int J Pharmacy Pharmaceut Sci.

[10] Jaradad NHF, Al Ali A (2015). Preliminary phytochemical screening, quantitative estimation of Total flavonoids, Total phenols and antioxidant activity of Ephedra Alata Decne. J Mater Environ Sci.

[11] Wagner HBS (1996). Plant drug analyses – a thin layer cromatography atlas, 2nd edn. Berlin, Heidelberg.

[12] Mosmann T (1983). Rapid colorimetric assay for cellular growth and survival: application to proliferation and cytotoxicity assays. J Immunol Methods.

[13] van Engeland M, Nieland LJW, Ramaekers FCS, Schutte B, Reutelingsperger CPM (1998). Annexin V-affinity assay: a review on an apoptosis detection system based on phosphatidylserine exposure. Cytometry.

[14] Vinci M, Gowan S, Boxall F, Patterson L, Zimmermann M, Court W, Lomas C, Mendiola M, Hardisson D, Eccles SA (2012). Advances in establishment and analysis of three-dimensional tumor spheroid-based functional assays for target validation and drug evaluation. BMC Biol.

[15] Vinci M, Box C, Zimmermann M, Eccles SA (2013). Tumor spheroid-based migration assays for evaluation of therapeutic agents. Methods Mol Biol.

[16] Holland PM, Abramson RD, Watson R, Gelfand DH (1991). Detection of specific polymerase chain reaction product by utilizing the 5′----3′ exonuclease activity of Thermus aquaticus DNA polymerase. Proc Natl Acad Sci U S A.

[17] Schmidt M, Scholz C, Gavril G, Otto C, Polednik C, Roller J, Hagen R (2014). Effect of Galium Verum aqueous extract on growth, motility and gene expression in drug-sensitive and -resistant laryngeal carcinoma cell lines. Int J Oncol.

[18] Singh A, Settleman J (2010). EMT, cancer stem cells and drug resistance: an emerging axis of evil in the war on cancer. Oncogene.

[19] Schmidt M, Polednik C, Roller J, Hagen R (2014). Galium Verum aqueous extract strongly inhibits the motility of head and neck cancer cell lines and protects mucosal keratinocytes against toxic DNA damage. Oncol Rep.

[20] Zhang X, Lin D, Jiang R, Li H, Wan J, Li H (2016). Ferulic acid exerts antitumor activity and inhibits metastasis in breast cancer cells by regulating epithelial to mesenchymal transition. Oncol Rep.

[21] Iizuka S, Ishimaru N, Kudo Y (2014). Matrix metalloproteinases: the gene expression signatures of head and neck cancer progression. Cancers.

[22] Tan Ide A, Ricciardelli C, Russell DL (2013). The metalloproteinase ADAMTS1: a comprehensive review of its role in tumorigenic and metastatic pathways. Int J Cancer.

[23] Lu X, Wang Q, Hu G, Van Poznak C, Fleisher M, Reiss M, Massague J, Kang Y (2009). ADAMTS1 and MMP1 proteolytically engage EGF-like ligands in an osteolytic signaling cascade for bone metastasis. Genes Dev.

[24] Kuang J, Zhao M, Li H, Dang W, Li W (2016). Identification of potential therapeutic target genes and mechanisms in head and neck squamous cell carcinoma by bioinformatics analysis. Oncol Lett.

[25] Lee J, Kim SS (2010). Current implications of cyclophilins in human cancers. J Exp Clin Cancer Res.

[26] Jayachandran A, Anaka M, Prithviraj P, Hudson C, McKeown SJ, Lo PH, Vella LJ, Goding CR, Cebon J, Behren A (2014). Thrombospondin 1 promotes an aggressive phenotype through epithelial-to-mesenchymal transition in human melanoma. Oncotarget.

[27] Roberts DD (1996). Regulation of tumor growth and metastasis by thrombospondin-1. FASEB J.

[28] Lowy CM, Oskarsson T, Tenascin C (2015). In metastasis: a view from the invasive front. Cell Adhes Migr.

[29] Mitsui Y, Shiina H, Kato T, Maekawa S, Hashimoto Y, Shiina M, Imai-Sumida M, Kulkarni P, Dasgupta P, Wong RK (2017). Versican promotes tumor progression, metastasis and predicts poor prognosis in renal carcinoma. Mol Cancer Res.

[30] Arellano-Garcia ME, Li R, Liu X, Xie Y, Yan X, Loo JA, Hu S (2010). Identification of tetranectin as a potential biomarker for metastatic oral cancer. Int J Mol Sci.

[31] Yang GW, Jiang JS, Lu WQ (2015). Ferulic acid exerts anti-Angiogenic and anti-tumor activity by targeting fibroblast growth factor receptor 1-mediated angiogenesis. Int J Mol Sci.

